# Regulation of root hair cell differentiation by R3 MYB transcription factors in tomato and *Arabidopsis*

**DOI:** 10.3389/fpls.2014.00091

**Published:** 2014-03-13

**Authors:** Rumi Tominaga-Wada, Takuji Wada

**Affiliations:** Graduate School of Biosphere Sciences, Hiroshima UniversityHigashi-Hiroshima, Japan

**Keywords:** *Arabidopsis*, MYB, root-hair, tomato, transcription factors

## Abstract

*CAPRICE* (*CPC*) encodes a small protein with an R3 MYB motif and regulates root hair and trichome cell differentiation in *Arabidopsis thaliana.* Six additional CPC-like MYB proteins including TRIPTYCHON (TRY), ENHANCER OF TRY AND CPC1 (ETC1), ENHANCER OF TRY AND CPC2 (ETC2), ENHANCER OF TRY AND CPC3/CPC-LIKE MYB3 (ETC3/CPL3), TRICHOMELESS1 (TCL1), and TRICHOMELESS2/CPC-LIKE MYB4 (TCL2/CPL4) also have the ability to regulate root hair and/or trichome cell differentiation in *Arabidopsis*. In this review, we describe our latest findings on how CPC-like MYB transcription factors regulate root hair cell differentiation. Recently, we identified the tomato *SlTRY* gene as an ortholog of the *Arabidopsis* *TRY* gene. Transgenic *Arabidopsis* plants harboring *SlTRY* produced more root hairs, a phenotype similar to that of *35S*::*CPC* transgenic plants. CPC is also known to be involved in anthocyanin biosynthesis. Anthocyanin accumulation was repressed in the *SlTRY* transgenic plants, suggesting that *SlTRY* can also influence anthocyanin biosynthesis. We concluded that tomato and *Arabidopsis* partially use similar transcription factors for root hair cell differentiation, and that a CPC-like R3 MYB may be a key common regulator of plant root-hair development.

## BRIEF BACKGROUND

**FIGURE 1:**
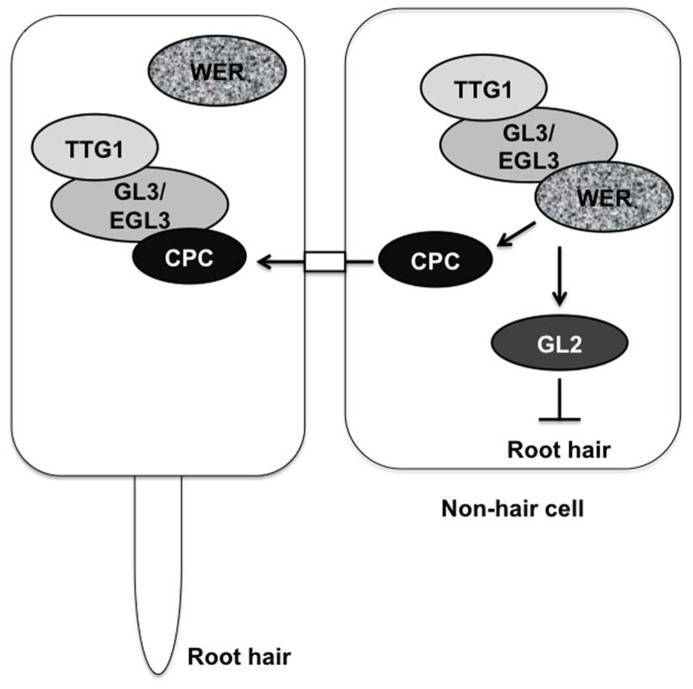
**Regulation of root-hair cell and non-hair cell fate determination by transcription factors.** The TTG1-GL3/EGL3-WER complex promotes *GL2* and *CPC* expression. The cell expressing *GL2* differentiates into a non-hair cell. The CPC protein moves from the non-hair cell to a neighboring cell and competes with WER for binding to GL3/EGL3. The TTG1-GL3/EGL3-CPC complex cannot promote *GL2* expression, resulting in the differentiation of a root-hair cell.

Cell fate determination is a critical step in plant development. In growing roots, epidermal cells differentiate into two cell types, root-hair cells, and non-hair cells in a file-specific manner. In *Arabidopsis* roots, epidermal cells in eight symmetrically positioned files differentiate into root-hair cells, and the cells of the other files become non-hair cells. Morphological analysis has shown the positional relationship between cortical cells and epidermal cells. Epidermal cells in contact with the junction of two underlying cortical cells differentiate into root-hair cells, whereas the cells in contact with only one cortical cell differentiate into non-hair cells (Dolan et al., 1993, 1994; Galway et al., 1994; Berger et al., 1998). Several regulatory factors are involved in root-hair or non-hair cell differentiation. The *glabra 2* (*gl2*) and *werewolf* (*wer*) mutants convert non-hair cells to root hair cells (Masucci et al., 1996; Lee and Schiefelbein, 1999). The *GL2* gene encodes a homeodomain leucine-zipper protein, and the *WER* gene encodes an R2R3-type MYB transcription factor that activates *GL2* expression preferentially in differentiating non-hair cells (Rerie et al., 1994; Di Cristina et al., 1996; Masucci et al., 1996; Lee and Schiefelbein, 1999). *GLABRA3* (*GL3*) and *ENHANCER OF GLABRA3* (*EGL3*) encode basic helix-loop-helix (bHLH) transcription factors that affect non-hair cell differentiation in a redundant manner, as evidenced by the conversion of non-hair cells to root-hair cells in the *gl3 egl3* double mutant (Bernhardt et al., 2003). Although, obvious increase in the number of root-hair cells was hardly observed in both *gl3* and *egl3* single mutants (Bernhardt et al., 2003). The *TRANSPARENT TESTA GLABRA1* (*TTG1*) gene is also involved in non-hair cell fate determination, as shown by the conversion of non-hair cells to root-hair cells in the *ttg1* mutant (Galway et al., 1994). The *TTG1* gene encodes a WD40-repeat protein (Walker et al., 1999). GL3 and EGL3 interact with WER (Bernhardt et al., 2003) and with TTG1 (Payne et al., 2000; Esch et al., 2003; Zhang et al., 2003) in yeast cells. A protein complex including WER, GL3/EGL3, and TTG1 acts upstream of the *GL2* gene in the root-hair regulatory pathway and promotes *GL2* gene expression (Galway et al., 1994; Rerie et al., 1994; Wada et al., 1997; Hung et al., 1998; Lee and Schiefelbein, 1999; Bernhardt et al., 2003, 2005). The cells expressing *GL2* differentiate into non-hair cells (**Figure 1**). In contrast, the root-hair cell differentiation is controlled by *CAPRICE* (*CPC*) as shown by a few root-hair phenotype of the *cpc* mutant (Wada et al., 1997). The *CPC* gene encodes R3-type MYB protein (Wada et al., 1997). The TTG1-GL3/EGL3-WER protein complex also up-regulates *CPC* gene expression in non-hair cells (Koshino-Kimura et al., 2005). The CPC protein moves from non-hair cells to neighboring cells and disturbs the formation of the TTG1-GL3/ETC3-WER transcriptional complex by competitively binding with WER (Wada et al., 2002; Koshino-Kimura et al., 2005; Kurata et al., 2005; Tominaga et al., 2007). The formation of the TTG1-GL3/EGL3-CPC protein complex represses expression of *GL2*, thereby inhibiting non-hair cell differentiation**(Wada et al., 2002; Kurata et al., 2005; **Figure 1**).

## THE* CPC* FAMILY PROMOTES ROOT-HAIR CELL DIFFERENTIATION

*CAPRICE* encodes a small protein with an R3 MYB motif and strongly promotes root-hair cell differentiation in *Arabidopsis* (Wada et al., 1997). In addition, we presented a model in which CPC was proposed to have evolved from WER (Tominaga et al., 2007). Chimeric constructs made from the R3 MYB regions of CPC and WER and used in reciprocal complementation tests revealed that the CPC R3 could not functionally substitute for WER R3 in the differentiation of non-hair cells (Tominaga et al., 2007). In contrast, WER R3 can substitute for CPC R3 (Tominaga et al., 2007). Our results suggest that CPC evolved from WER after truncation of the activation domain and loss of DNA binding ability (Tominaga et al., 2007). *Arabidopsis* has six additional *CPC*-like MYB sequences in its genome, including *TRY*, *ETC1*, *ETC2*, *ETC3/CPL3*, *TCL1*, and *TCL2/CPL4* (Hulskamp et al., 1994; Schellmann et al., 2002; Esch et al., 2004; Kirik et al., 2004a,b; Simon et al., 2007; Wang et al., 2007, 2008, 2010; Tominaga et al., 2008; Wester et al., 2009; Gan et al., 2011; Tominaga-Wada and Nukumizu, 2012). These seven CPC-like MYB transcription factors act as positive regulators of root-hair cell differentiation and as negative regulators of trichome differentiation in a partially redundant manner (Tominaga-Wada et al., 2011; Tominaga-Wada and Nukumizu, 2012). The *try* mutant forms trichome clusters on leaves indicating that TRY functions in trichome differentiation (Hulskamp et al., 1994; Schellmann et al., 2002). *ETC1* and *ETC2* have redundant and enhancer functions with *CPC* and *TRY* in root-hair and trichome differentiation (Esch et al., 2004; Kirik et al., 2004a,b). Therefore, these genes were named *ENHANCER OF TRY AND CPC* (*ETC*; Esch et al., 2004; Kirik et al., 2004a,b). *TCL1* and *TCL2 *negatively regulate trichome formation on the inflorescence stems and pedicels (Wang et al., 2007; Gan et al., 2011). These findings suggest functional divergence among *CPC* family genes.

## RECENT FINDINGS ON THE FUNCTIONS OF THE *CPC* FAMILY

We have identified the *CPL4* gene between At2g30430 and *ETC2* (At2g30420) independently of Gan et al. (Gan et al., 2011; Tominaga-Wada and Nukumizu, 2012). Between *CPL4* and *ETC2*, there were several chimeric transcripts generated through alternative splicing (Tominaga-Wada and Nukumizu, 2012). Our study proposed that inter-genic alterative splicing also characterizes the CPC-like MYB gene family (Tominaga-Wada and Nukumizu, 2012).

A lateral inhibition mechanism mediated by cell-to-cell movement of CPC was thought to cause cell fate specification (Lee and Schiefelbein, 2002; Kwak and Schiefelbein, 2007, 2008). However, it is unclear how *CPC*, which is preferentially expressed in non-hair cells, specifically acts in the root-hair cells rather than in non-hair cells. Recently, nuclear trapping of CPC in the root-hair cells by EGL3 was suggested to be involved in root-hair cell differentiation (Kang et al., 2013). CPC protein accumulates predominantly in the nuclei of root-hair cells in the early meristematic region, and this localization requires specific expression of *EGL3* in the root-hair cells (Kang et al., 2013). These results suggest that cell-to-cell movement of CPC occurs within the meristem of root epidermal cells and that EGL3 traps the CPC protein in the root-hair cells (Kang et al., 2013). CPC and TRY were reported to recruit AtMYC1 into the nucleus, suggesting mutual control of the intracellular localization of patterning proteins (Pesch et al., 2013). *AtMYC1*, a homologue of *GL3* and *EGL3*, encodes a bHLH transcription factor predominantly localized in the cytoplasm (Urao et al., 1996; Pesch et al., 2013). AtMYC1 regulates the distribution of GL1 protein between the nucleus and the cytoplasm. On the other hand, AtMYC1 is recruited into the nucleus by TRY and CPC, subsequent to significant accumulation of TRY and CPC in the nucleus (Pesch et al., 2013). These results and genetic analyses imply that AtMYC1 represses the activity of TRY and CPC (Pesch et al., 2013).

Tissue-specific transcript profiling also indicated that there were some redundancies between *CPC* and *TRY* at the transcriptional level (Simon et al., 2013). We have extended the characterization of CPC-like MYB genes to include the identification of inter-genic alterative splicing and precise expression patterns using tissue-specific transcript profiling (Tominaga-Wada and Nukumizu, 2012; Simon et al., 2013). Recent findings have also revealed that in addition to the formation of the transcription complex, each type of transcription factor can regulate the inter- and intra-cellular localization of the other types to regulate root hair and trichome formation (Kang et al., 2013; Pesch et al., 2013).

## A *CPC*-LIKE MYB IN TOMATO

**FIGURE 2:**
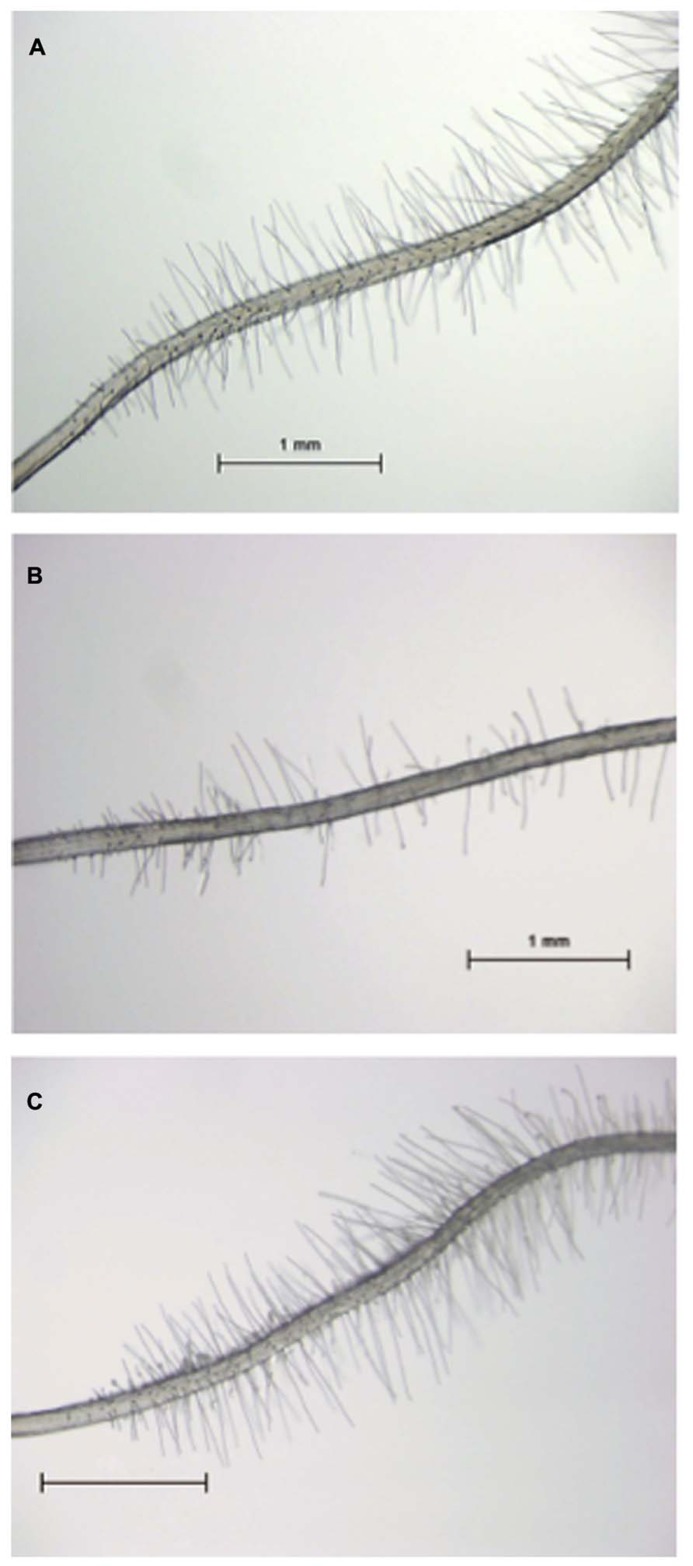
**Root-hair phenotype of *CPC*::*SlTRY* transgenic plants. (A)** Root-hair phenotype of a 5-day-old *Arabidopsis* wild type (Col-0) seedling. **(B)** Root-hair phenotype of a 5-day-old *Arabidopsis* *cpc-2* mutant seedling. **(C)** Root-hair phenotype of a 5-day-old *Arabidopsis* *cpc-2* mutant seedling transformed with *CPC*::*SlTRY*. Scale bars: 1 mm.

Recently, we identified the tomato *SlTRY* gene as an ortholog of an *Arabidopsis* *CPC*-like MYB gene (Tominaga-Wada et al., 2013b). The *CPC*::*SlTRY* construct in *cpc-2* transgenic plants increased the number of root-hairs compared with that of the *cpc-2* mutant plants (**Figure 2**; Tominaga-Wada et al., 2013b). These results suggest that tomato and *Arabidopsis* use common transcription factors for root-hair differentiation. In addition to root-hair cell differentiation, the *Arabidopsis* *CPC* gene is known to regulate anthocyanin biosynthesis (Zhu et al., 2009). Anthocyanin accumulation was repressed in the *CPC*::*SlTRY* transgenic plants as was observed in the *35S*::*CPC* transgenic plants, suggesting that *SlTRY* also influences anthocyanin pigment synthesis (Tominaga-Wada et al., 2013a). Tomato and *Arabidopsis* partially use similar transcription factors for root hair cell differentiation, and a CPC-like R3 MYB may be a key common regulator of plant root-hair development. Further analysis of CPC-like gene function in tomato is on-going.

## FUTURE PERSPECTIVES

The cell-to-cell movement of CPC from non-hair cells to root-hair cells is important for root-hair cell specification; however, the precise mechanism of CPC movement is unknown. How CPC is targeted, transported through plasmodesmata, and trapped in the nucleus of the root-hair cells to define cell fate is an intriguing problem.

Transcriptome analyses provide detailed characterizations of transcription factors involved in root epidermal cell differentiation. Further molecular characterization of individual genes and mutant phenotypes is necessary to fully assess the precise mechanism for root epidermal cell differentiation, including an analysis of redundancies in the epidermal cell regulatory pathway.

*TRY* and *GL3* homologous genes were identified from the tomato genome and named *SlTRY* and *SlGL3*, respectively (Tominaga-Wada et al., 2013b). *SlTRY* showed a similar function to *TRY*, including inhibition of trichome formation and enhancement of root-hair differentiation. On the other hand, *SlGL3* did not show any obvious effect on trichome or non-hair cell differentiation (Tominaga-Wada et al., 2013b). There may be other GL3 ortholog(s) in the unannotated tomato genomes, or tomato uses other pathways to regulate epidermal cell differentiation. Further studies to determine the functions of R3-MYB and bHLH in epidermal cell differentiation in tomato are required.

## Conflict of Interest Statement

The authors declare that the research was conducted in the absence of any commercial or financial relationships that could be construed as a potential conflict of interest.

## References

[B1] Berger F. Haseloff J. Schiefelbein J. Dolan L. (1998). Positional information in root epidermis is defined during embryogenesis and acts in domains with strict boundaries. *Curr. Biol.* 8 421–430 9550701 10.1016/s0960-9822(98)70176-9

[B2] Bernhardt C. Lee M. M. Gonzalez A. Zhang F. Lloyd A. Schiefelbein J. (2003). The bHLH genes GLABRA3 (GL3) and ENHANCER OF GLABRA3 (EGL3) specify epidermal cell fate in the *Arabidopsis* root. *Development* 130 6431–6439 10.1242/dev.00880 14627722

[B3] Bernhardt C. Zhao M. Gonzalez A. Lloyd A. Schiefelbein J. (2005). The bHLH genes GL3 and EGL3 participate in an intercellular regulatory circuit that controls cell patterning in the *Arabidopsis* root epidermis. *Development* 132 291–298 10.1242/dev.01565 15590742

[B4] Di Cristina M. Sessa G. Dolan L. Linstead P. Baima S. Ruberti I. (1996). The *Arabidopsis* Athb-10 (GLABRA2) is an HD-Zip protein required for regulation of root hair development. *Plant J.* 10 393–402 10.1046/j.1365-313X.1996.10030393.x 8811855

[B5] Dolan L. Duckett C. M. Grierson C. Linstead P. Schneider K. Lawson E. (1994). Clonal relationships and cell patterning in the root epidermis of *Arabidopsis*. *Development* 120 2465–2474

[B6] Dolan L. Janmaat K. Willemsen V. Linstead P. Poethig S. Roberts K. (1993). Cellular organisation of the *Arabidopsis thaliana* root. *Development* 119 71–84 8275865 10.1242/dev.119.1.71

[B7] Esch J. J. Chen M. A. Hillestad M. Marks M. D. (2004). Comparison of TRY and the closely related At1g01380 gene in controlling *Arabidopsis* trichome patterning. *Plant J.* 40 860–869 15584952 10.1111/j.1365-313X.2004.02259.x

[B8] Esch J. J. Chen M. Sanders M. Hillestad M. Ndkium S. Idelkope B. (2003). A contradictory GLABRA3 allele helps define gene interactions controlling trichome development in *Arabidopsis*. *Development* 130 5885–5894 10.1242/dev.00812 14561633

[B9] Galway M. E. Masucci J. D. Lloyd A. M. Walbot V. Davis R. W. Schiefelbein J. W. (1994). The TTG gene is required to specify epidermal cell fate and cell patterning in the *Arabidopsis* root. *Dev. Biol.* 166 740–754 7813791 10.1006/dbio.1994.1352

[B10] Gan L. Xia K. Chen J. G. Wang S. (2011). Functional Characterization of TRICHOMELESS2, a new single-repeat R3 MYB transcription factor in the regulation of trichome patterning in *Arabidopsis*. *BMC Plant Biol.* 11:176 10.1186/1471-2229-11-176 PMC3264604 22168948

[B11] Hulskamp M. Misra S. Jurgens G. (1994). Genetic dissection of trichome cell development in *Arabidopsis*. *Cell* 76 555–566 10.1016/0092-8674(94)90118-X 8313475

[B12] Hung C. Y. Lin Y. Zhang M. Pollock S. Marks M. D. Schiefelbein J. (1998). A common position-dependent mechanism controls cell-type patterning and GLABRA2 regulation in the root and hypocotyl epidermis of *Arabidopsis*. *Plant Physiol.* 117 73–84 10.1104/pp.117.1.73 9576776 PMC35023

[B13] Kang Y. H. Song S. K. Schiefelbein J. Lee M. M. (2013). Nuclear trapping controls the position-dependent localization of CAPRICE in the root epidermis of *Arabidopsis*. *Plant Physiol.* 163 193–204 10.1104/pp.113.221028 23832626 PMC3762640

[B14] Kirik V. Simon M. Huelskamp M. Schiefelbein J. (2004a). The ENHANCER OF TRY AND CPC1 gene acts redundantly with TRIPTYCHON and CAPRICE in trichome and root hair cell patterning in *Arabidopsis*. *Dev. Biol.* 268 506–513 15063185 10.1016/j.ydbio.2003.12.037

[B15] Kirik V. Simon M. Wester K. Schiefelbein J. Hulskamp M. (2004b). ENHANCER of TRY and CPC 2 (ETC2) reveals redundancy in the region-specific control of trichome development of *Arabidopsis*. *Plant Mol. Biol.* 55 389–398 10.1007/s11103-004-0893-8 15604688

[B16] Koshino-Kimura Y. Wada T. Tachibana T. Tsugeki R. Ishiguro S. Okada K. (2005). Regulation of CAPRICE transcription by MYB proteins for root epidermis differentiation in *Arabidopsis*. *Plant Cell Physiol.* 46 817–826 15795220 10.1093/pcp/pci096

[B17] Kurata T. Ishida T. Kawabata-Awai C. Noguchi M. Hattori S. Sano R. (2005). Cell-to-cell movement of the CAPRICE protein in *Arabidopsis* root epidermal cell differentiation. *Development* 132 5387–5398 16291794 10.1242/dev.02139

[B18] Kwak S. H. Schiefelbein J. (2007). The role of the SCRAMBLED receptor-like kinase in patterning the *Arabidopsis* root epidermis. *Dev. Biol.* 302 118–131 10.1016/j.ydbio.2006.09.009 17027738

[B19] Kwak S. H. Schiefelbein J. (2008). Cellular pattern formation by SCRAMBLED, a leucine-rich repeat receptor-like kinase in *Arabidopsis*. *Plant Signal. Behav.* 3 110–112 19704725 10.4161/psb.3.2.4969 PMC2633995

[B20] Lee M. M. Schiefelbein J. (1999). WEREWOLF, a MYB-related protein in *Arabidopsis*, is a position-dependent regulator of epidermal cell patterning. *Cell* 99 473–483 10.1016/S0092-8674(00)81536-6 10589676

[B21] Lee M. M. Schiefelbein J. (2002). Cell pattern in the *Arabidopsis* root epidermis determined by lateral inhibition with feedback. *Plant Cell* 14 611–618 10.1105/tpc.010434 11910008 PMC150583

[B22] Masucci J. D. Rerie W. G. Foreman D. R. Zhang M. Galway M. E. Marks M. D. (1996). The homeobox gene GLABRA2 is required for position-dependent cell differentiation in the root epidermis of *Arabidopsis thaliana*. *Development* 122 1253–1260 8620852 10.1242/dev.122.4.1253

[B23] Payne C. T. Zhang F. Lloyd A. M. (2000). GL3 encodes a bHLH protein that regulates trichome development in *Arabidopsis* through interaction with GL1 and TTG1. *Genetics* 156 1349–1362 11063707 10.1093/genetics/156.3.1349 PMC1461316

[B24] Pesch M. Schultheiss I. Digiuni S. Uhrig J. F. Hulskamp M. (2013). Mutual control of intracellular localisation of the patterning proteins AtMYC1, GL1 and TRY/CPC in *Arabidopsis*. *Development* 140 3456–3467 10.1242/dev.094698 23900543

[B25] Rerie W. G. Feldmann K. A. Marks M. D. (1994). The GLABRA2 gene encodes a homeo domain protein required for normal trichome development in *Arabidopsis*. *Genes Dev.* 8 1388–1399 7926739 10.1101/gad.8.12.1388

[B26] Schellmann S. Schnittger A. Kirik V. Wada T. Okada K. Beermann A. (2002). TRIPTYCHON and CAPRICE mediate lateral inhibition during trichome and root hair patterning in *Arabidopsis*. *EMBO J.* 21 5036–5046 10.1093/emboj/cdf524 12356720 PMC129046

[B27] Simon M. Bruex A. Kainkaryam R. M. Zheng X. Huang L. Woolf P. J. (2013). Tissue-specific profiling reveals transcriptome alterations in *Arabidopsis* mutants lacking morphological phenotypes. *Plant Cell* 25 3175–3185 10.1105/tpc.113.115121 24014549 PMC3809526

[B28] Simon M. Lee M. M. Lin Y. Gish L. Schiefelbein J. (2007). Distinct and overlapping roles of single-repeat MYB genes in root epidermal patterning. *Dev. Biol.* 311 566–578 10.1016/j.ydbio.2007.09.001 17931617

[B29] Tominaga R. Iwata M. Okada K. Wada T. (2007). Functional analysis of the epidermal-specific MYB genes CAPRICE and WEREWOLF in *Arabidopsis*. *Plant Cell* 19 2264–2277 10.1105/tpc.106.045732 17644729 PMC1955706

[B30] Tominaga R. Iwata M. Sano R. Inoue K. Okada K. Wada T. (2008). *Arabidopsis* CAPRICE-LIKE MYB 3 (CPL3) controls endoreduplication and flowering development in addition to trichome and root hair formation. *Development* 135 1335–1345 10.1242/dev.017947 18305006

[B31] Tominaga-Wada R. Nukumizu Y. (2012). Expression analysis of an R3-type MYB transcription factor CPC-LIKE MYB4 (TRICHOMELESS2) and CPL4-related transcripts in *Arabidopsis*. *Int. J. Mol. Sci.* 13 3478–3491 10.3390/ijms13033478 22489163 PMC3317723

[B32] Tominaga-Wada R. Ishida T. Wada T. (2011). New insights into the mechanism of development of *Arabidopsis* root hairs and trichomes. *Int. Rev. Cell Mol. Biol.* 286 67–106 10.1016/B978-0-12-385859-7.00002-1 21199780

[B33] Tominaga-Wada R. Nukumizu Y. Wada T. (2013a). Tomato (*Solanum lycopersicum*) homologs of TRIPTYCHON (SlTRY) and GLABRA3 (SlGL3) are involved in anthocyanin accumulation. *Plant Signal. Behav.* 8:e24575 10.4161/psb.24575 PMC3907391 23603939

[B34] Tominaga-Wada R. Nukumizu Y. Sato S. Wada T. (2013b). Control of plant trichome and root-hair development by a tomato (*Solanum lycopersicum*) R3 MYB transcription factor. *PLoS ONE* 8:e54019 10.1371/journal.pone.0054019 PMC3543402 23326563

[B35] Urao T. Yamaguchi-Shinozaki K. Mitsukawa N. Shibata D. Shinozaki K. (1996). Molecular cloning and characterization of a gene that encodes a MYC-related protein in *Arabidopsis*. *Plant Mol. Biol.* 32 571–576 10.1007/BF00019112 8980509

[B36] Wada T. Kurata T. Tominaga R. Koshino-Kimura Y. Tachibana T. Goto K. (2002). Role of a positive regulator of root hair development, CAPRICE, in *Arabidopsis* root epidermal cell differentiation. *Development* 129 5409–5419 12403712 10.1242/dev.00111

[B37] Wada T. Tachibana T. Shimura Y. Okada K. (1997). Epidermal cell differentiation in *Arabidopsis* determined by a Myb homolog, CPC. *Science* 277 1113–1116 10.1126/science.277.5329.1113 9262483

[B38] Walker A. R. Davison P. A. Bolognesi-Winfield A. C. James C. M. Srinivasan N. Blundell T. L. (1999). The TRANSPARENT TESTA GLABRA1 locus, which regulates trichome differentiation and anthocyanin biosynthesis in *Arabidopsis*, encodes a WD40 repeat protein. *Plant Cell* 11 1337–1350 10.1105/tpc.11.7.1337 10402433 PMC144274

[B39] Wang S. Barron C. Schiefelbein J. Chen J. G. (2010). Distinct relationships between GLABRA2 and single-repeat R3 MYB transcription factors in the regulation of trichome and root hair patterning in *Arabidopsis*. *New Phytol.* 185 387–400 10.1111/j.1469-8137.2009.03067.x 19878461

[B40] Wang S. Hubbard L. Chang Y. Guo J. Schiefelbein J. Chen J. G. (2008). Comprehensive analysis of single-repeat R3 MYB proteins in epidermal cell patterning and their transcriptional regulation in *Arabidopsis*. *BMC Plant Biol.* 8:81 10.1186/1471-2229-8-81 PMC2492867 18644155

[B41] Wang S. Kwak S. H. Zeng Q. Ellis B. E. Chen X. Y. Schiefelbein J. (2007). TRICHOMELESS1 regulates trichome patterning by suppressing GLABRA1 in *Arabidopsis*. *Development* 134 3873–3882 10.1242/dev.009597 17933793

[B42] Wester K. Digiuni S. Geier F. Timmer J. Fleck C. Hulskamp M. (2009). Functional diversity of R3 single-repeat genes in trichome development. *Development* 136 1487–1496 10.1242/dev.021733 19336467

[B43] Zhang F. Gonzalez A. Zhao M. Payne C. T. Lloyd A. (2003). A network of redundant bHLH proteins functions in all TTG1-dependent pathways of *Arabidopsis*. *Development* 130 4859–4869 10.1242/dev.00681 12917293

[B44] Zhu H. F. Fitzsimmons K. Khandelwal A. Kranz R. G. (2009). CPC, a single-repeat R3 MYB, is a negative regulator of anthocyanin biosynthesis in *Arabidopsis*. *Mol. Plant* 2 790–802 10.1093/mp/ssp030 19825656

